# Biomechanical analyses of common suspension sutures in primary cleft lip rhinoplasty

**DOI:** 10.1186/s13005-019-0203-y

**Published:** 2019-07-17

**Authors:** Hanyao Huang, Xu Cheng, Xiangyou Luo, Bing Shi, Jingtao Li

**Affiliations:** State Key Laboratory of Oral Diseases & National Clinical Research Center for Oral Diseases & Department of Oral Maxillofacial Surgery, West China Hospital of Stomatology, Sichuan University, 14 Renmin South Road, Chengdu, 610041 China

**Keywords:** Cleft lip, Nasal deformity, Rhinoplasty, Finite element analysis, Biomechanics

## Abstract

**Background:**

For a better understanding of common suspension sutures during primary cleft lip nasal rhinoplasty, the biomechanical consequences of those sutures need to be demonstrated.

**Methods:**

A finite element model of the infant specimen was established. The closure of cleft lip and four different specific suspension sutures were simulated by loading different forces on the model: 1. F1 to simulate the suture fastening both medial crura together; 2. F2 to simulate the suture which sewed both medial crura and the non-cleft-side upper lateral cartilage together; 3. F3 to simulate the suture elevating the alar cartilage cranially; 4. F4 to simulate the suture elevating the alar cartilage superiorly. The deformation and stress distribution consequent to each maneuver were analyzed in details.

**Results:**

The deviation of columella was restored through the closure of cleft lip. Different suspension sutures had different biomechanical effects on the nasal structure. All suspension sutures had the function on elevating the alar cartilage. F2 had no function on restoring the collapse of the nasal tip. The suture which fastened both medial crura together leaded to the lowest stress on the skin envelope.

**Conclusions:**

Each suspension suture had its characteristics respectively. The simulation suggested that F1, the suture which fastened both medial crura, could be the most potential maneuver for cleft lip rhinoplasty because it can symmetrically restore the shape of the nose without incurring a significant increase in stress.

## Background

Cleft lip deformity is a most common congenital craniofacial defect in human [1], while the cleft lip nasal deformity is one of the greatest challenges to cleft surgeons [2, 3]. Different surgical maneuvers have been applied to restore the anatomical nasal structure, but the outcomes were indistinguishable because most of the analyses for surgical outcomes were retrospective and the measurement criteria were arduous to unify. Choosing an appropriate maneuver guarantees the favorable surgery effect. The biomechanics for cleft lip nose correction and a better understanding of different surgical maneuvers should be considered as the primary requirement.

Early physical simulations illustrated that the cartilages framework was correlated to the shape of the nose, but the mechanisms were not demonstrated specifically [4]. For understanding the biomechanics of rhinoplasty better, finite element analyses have been validated as a rewarding method [5–13]. Our recent studies demonstrated the necessary maneuvers in a competent cleft lip rhinoplasty [12], and relapse would occur according to selected surgical techniques [11].

Suspension sutures have been widely applied to cleft lip rhinoplasty [14–17]. However, they are varied from different medical centers for cleft care. It is formidable to decide which suture is most available due to the poor biomechanical understanding of those maneuvers. Each suture must have its mechanism for repairing the nasal deformity. A comprehensive understanding of the deformation and stress generated by each specific surgical maneuver would be of great value for choosing the most appropriate corrective technique.

In this study, a finite element model of complete unilateral cleft lip nasal deformity was established to demonstrate the biomechanical consequences of those common suspension sutures, including the methods of Millard, Cutting, McComb and Noordhoff [14–17], during primary cleft lip nasal rhinoplasty. The closure of cleft lip and different suspension sutures during rhinoplasty were simulated. Their corresponding morphological deformation and stress distribution were well demonstrated in details.

## Material and methods

**Fig. 1:**
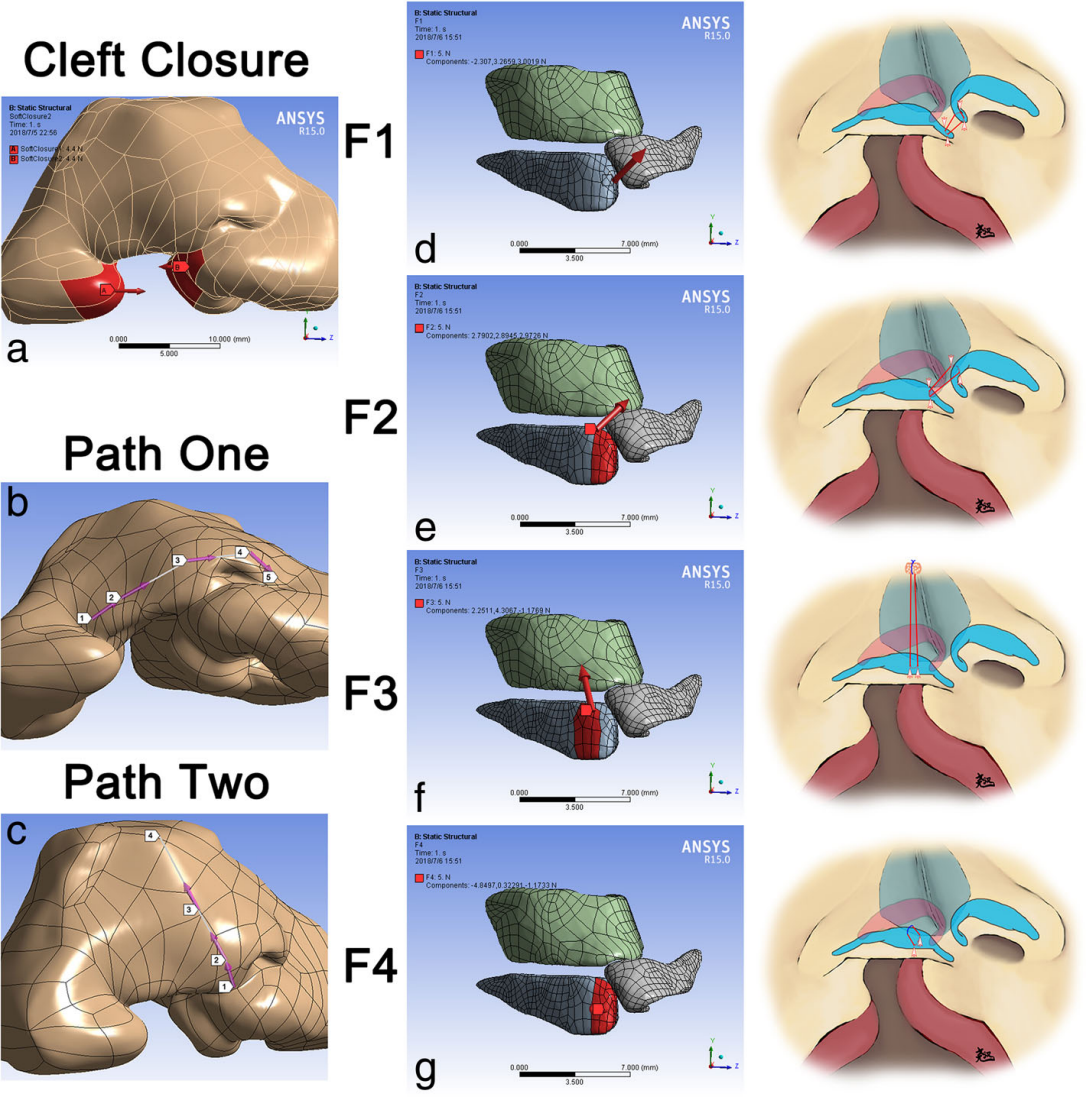
Vectors of force loadings and definition of paths. **a** Two opposite forces at both sides of the cleft to simulate the closure of cleft lip; **b** Path one was defined by the alar bases at both sides (Landmarks one, five), the alar domes at both sides (Landmark two, four) and the nasal tip (Landmark three); **c** Path two was defined by the columella base (Landmark one), the nasal tip (Landmark two), the dorsum (Landmark three) and the nasal radix (Landmark four); **d** Medially, anteriorly and cranially directed force on the tip of medial crus (F1) to simulate the suture fastened both medial crura together; **e** Medially, posteriorly and cranially directed force on the medial crus (F2) to simulate the suture sewing both medial crura and the non-cleft-side upper lateral cartilage together; **f** Force directed to the nasal radix and paralleled to the dorsum on the intermediate crus (F3) to simulate the suture elevating the alar cartilage cranially; **g** Anteriorly directed force on the intermediate crus (F4) to simulate the suture elevating the alar cartilage superiorly. (**b**, **c**, **d**, **e** Right) Surgical maneuvers illustration for each force. Red line, suture inside the body. Blue line, extracorporeal suture

Research specimen, nasal model reconstruction and boundary condition were described in our published paper [12]. In this study, the finite element model was established basing on the micro-MRI of an infant specimen with unilateral cleft lip nasal deformity. The nasal cartilage framework included the alar cartilages, the upper lateral cartilages and the nasal septum, which could be differentiated from the surrounding tissue by micro-MRI. As the muscles and skins of the nasal system were not specified in our model, in which the soft tissues were regarded as a whole, the boundary condition between the cartilages and soft tissues were set as attached. The closure of cleft lip (Fig. 1a) and four different specific suspension sutures were simulated (Fig. 1DEFG). Two paths on the cutaneous surface of the skin envelope were applied for demonstrating the total deformation and the equivalent von-mises stress at critical nasal landmarks (Fig. 1 b, c).

For simulating the closure of cleft on the soft envelope, the magnitudes of two opposite forces at both sides of the cleft were increased as the same from 0 N to the value for exactly closing the cleft (Fig. 1a).

To simulate the functions of different suspension sutures in primary cleft lip rhinoplasty, the forces were loaded on the alar cartilage at the cleft side in four different vectors after the closure of the cleft lip: 1. Millard’s method: Medially, anteriorly and cranially directed force on the tip of medial crus (F1) (Fig. 1d); 2. Cutting’s method: Medially, posteriorly and cranially directed force on the medial crus (F2) (Fig. 1e); 3. McComb’s method: Force directed to the nasal radix and paralleled to the dorsum on the intermediate crus (F3) (Fig. 1f); 4. Noordhoff’s method: Anteriorly directed force on the intermediate crus (F4) (Fig. 1g). The magnitudes of these four forces were 5 N.

Static structural analysis was applied to calculate outcomes. The total deformation (TD) and equivalent von-mises stress (EQV) of the model were scaled in millimeter and kPa.

## Results

### The deviation of columella was restored through the closure of cleft lip

**Fig. 2:**
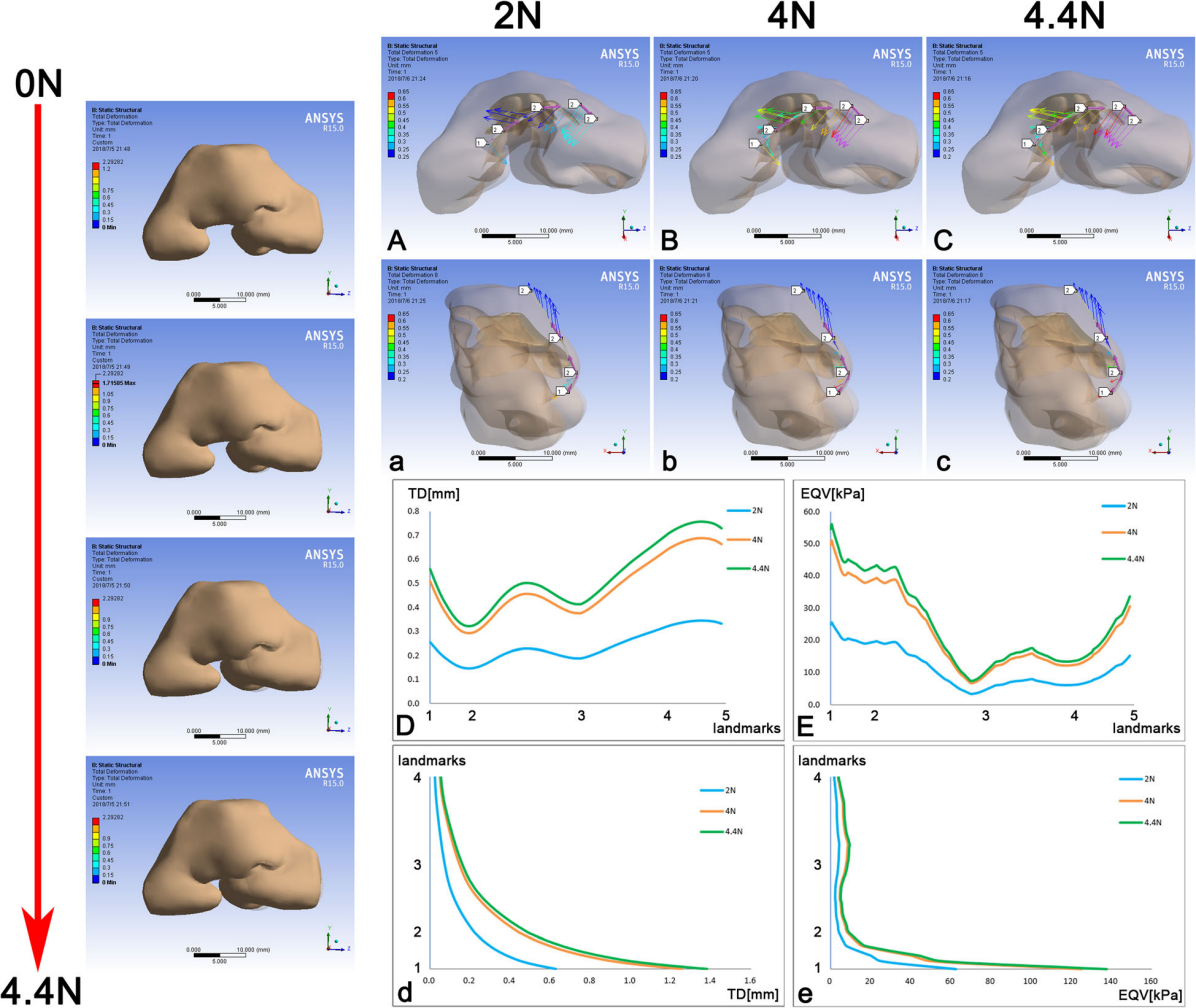
TD and EQV of the skin envelope and major landmarks consequent to the closure of cleft lip. The magnitudes of two opposite forces at both sides of the cleft were as the same to generate the TD. The whole process of force loading was demonstrated on the left, from top to bottom respectively in 0 N, 2 N, 4 N and 4,4 N which closed the cleft exactly. **A**, **B**, **C** The vectors of TD on Path one; **a**, **b**, **c** The vectors of TD on Path two; **D** The TD on Path one; **E** The EQV on Path one; **d** The TD on Path two; **e** The EQV on Path two. Path one was defined by the alar bases at both sides (Landmarks one, five), the alar domes at both sides (Landmark two, four) and the nasal tip (Landmark three); Path two was defined by the columella base (Landmark one), the nasal tip (Landmark two), the dorsum (Landmark three) and the nasal radix (Landmark four). The grey shadow represented the pre-simulation position of the model. TD, total deformation; EQV, equivalent von-mises stress

The cleft on the skin envelope which represented the cleft lip was first closed. The model before force loading served as the control. The morphology of the whole model changed and the width of cleft decreased with the magnitudes of both forces increased gradually. The total deformation of the skin envelope at 2 N and 4 N were shown (Fig. 2). When width came to zero, the magnitudes were 4.4 N (Fig. 2).

When closing the cleft lip, the nasal morphology would change because the nasolabial structure was considered as a system that cannot be separated. Two paths on the cutaneous surface were applied to specify the outcomes on landmarks. Effect of the skin envelope mainly concentrated on the horizontal plane instead of the sagittal plane (Fig. 2ABCabc). On the horizontal plane, deformation at the non-cleft side was more obvious (Fig. 2D) and pointed medially and posteriorly (Fig. 2ABC). The deformation between the nasal tip and the dome pointed laterally, as the deformation between the dome and the alar base at the cleft side point medially and posteriorly (Fig. 2ABC). With the closure of the cleft lip, only the deformation around the columella base was shown along the sagittal plane (Fig. 2abcd). The equivalent von-mises stresses (EQV) on two paths were almost the same patterns as the TD along the paths (Fig. 2Ee). Therefore, the lateral displacement of the alar base and the deviation of columella were restored when closing the cleft lip, but the collapse of the nasal tip became even worse than before.

### Different suspension sutures had different biomechanical effects on the nasal structure

The suspension sutures for primary nasal deformity among patients with unilateral cleft lip consisted of four maneuvers, which were simulated by F1 (Millard), F2 (Cutting), F3 (McComb) and F4 (Noordhoff) respectively.

**Fig. 3:**
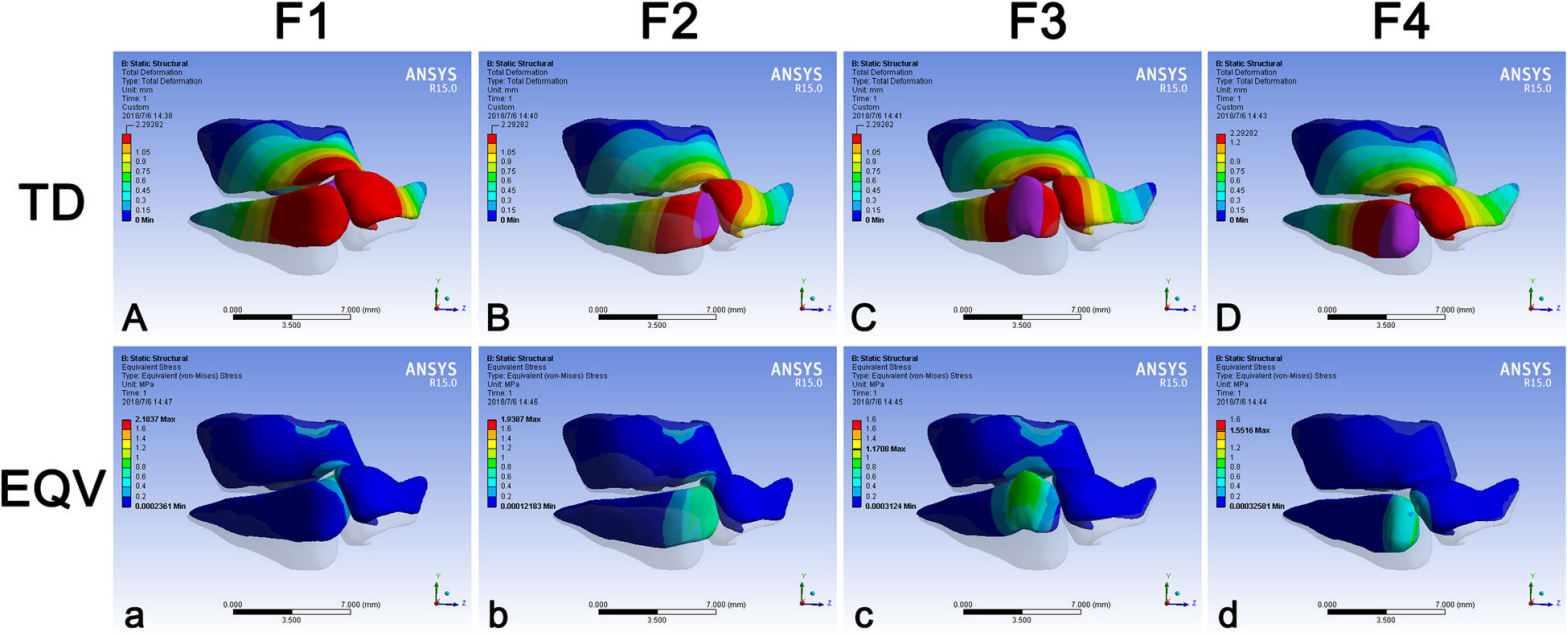
TD and EQV of the nasal cartilage framework consequent to different force loadings. The TD (mm) and EQV (kPa) of the nasal cartilage framework were demonstrated under F1 (**Aa**), F2 (**Bb**), F3 (**Cc**) and F4 (**Dd**). Blue represented the stable part of the model. The changes of TD and EQV were corresponding to color change. The grey shadow represented the pre-simulation position of the model. TD, total deformation; EQV, equivalent von-mises stress

All force had the function on elevating the alar cartilage. The elevation by F3 was the most significant (Fig. 3ABCD). When loading F1 and F2, two alar cartilages became closed, and the whole cartilage framework tended to move to the non-cleft side (Fig. 3AB). When loading F4, except the loaded alar cartilage, the other parts of the cartilage framework were less influenced compared to the other three forces (Fig. 3Dd). The stress of all models concentrated on the location where the force was loaded, and only F4 had no visible effect on the upper cartilage (Fig. 3abcd).

**Fig. 4:**
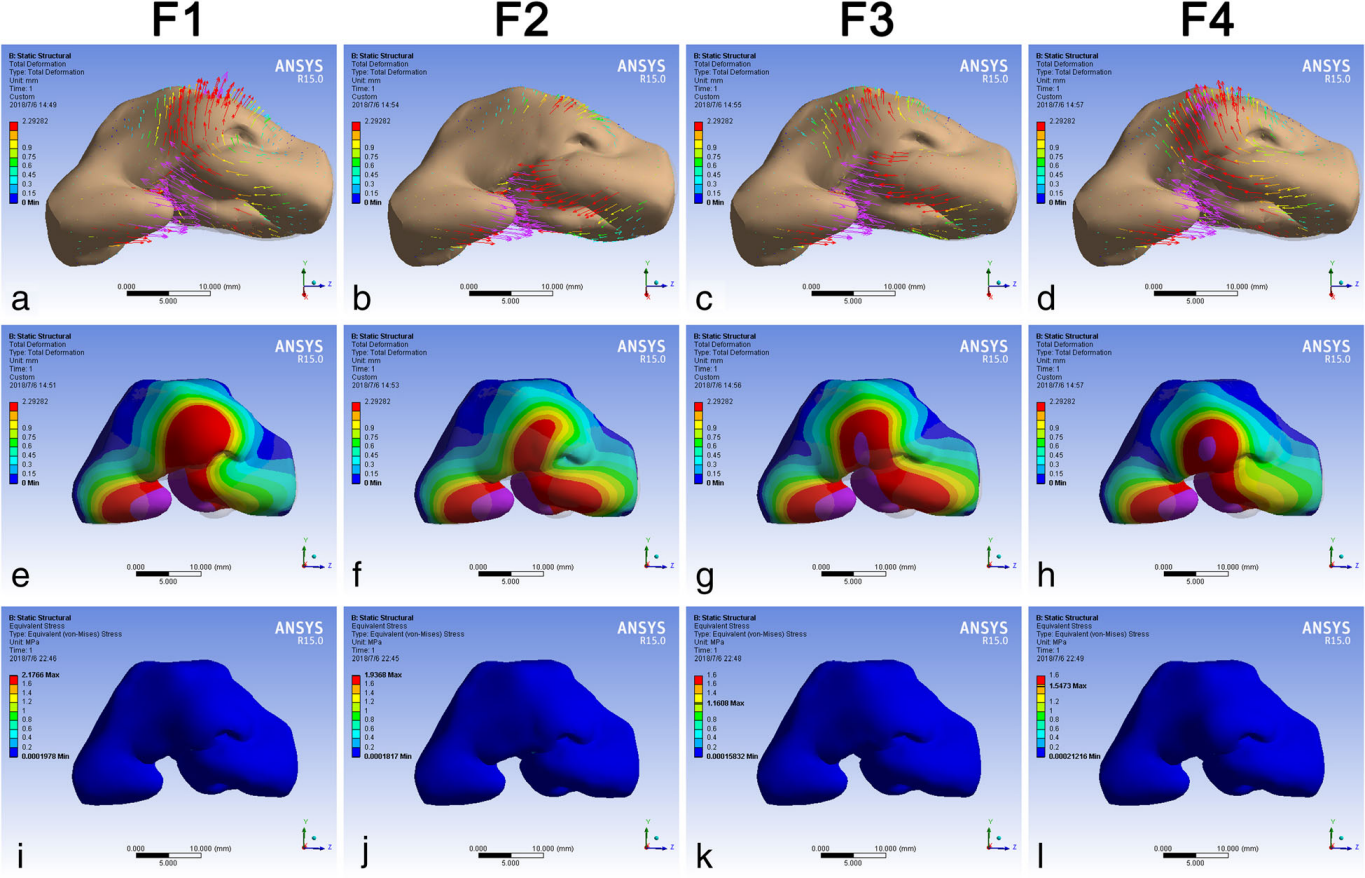
TD and EQV of the skin envelope consequent to different force loadings. The TD (mm) of the skin envelope was demonstrated under F1 (**a**, **e**), F2 (**b**, **f**), F3 (**c**, **g**) and F4 (**d**, **h**). The EQV (kPa) of the skin envelope was demonstrated under F1 (**i**), F2 (**j**), F3 (**k**) and F4 (**l**). Blue represented the stable part of the model. The length and direction of the arrow represented the value and direction of the deformation respectively. The changes of TD and EQV were corresponding to color change. The grey shadow represented the pre-simulation position of the model. TD, total deformation; EQV, equivalent von-mises stress

All maneuvers had the function on restoring the collapse of the nasal tip except F2 (Fig. 4a, b, c, d). The deviation of the whole nasal system was enhanced by F1 and F2 (Fig. 4a, b). When loading F3 and F4, however, the deviation could be restored (Fig. 4c, d). The ranges of TD on the skin envelope caused by four forces were almost the same, from the nasal radix to areas around the alar bases at both sides (Fig. 4e, f, g, h). The EQV on the skin envelope, however, was only observed at the columella base under each circumstance and was overwhelmingly minor (Fig. 4i, j, k, l).

**Fig. 5:**
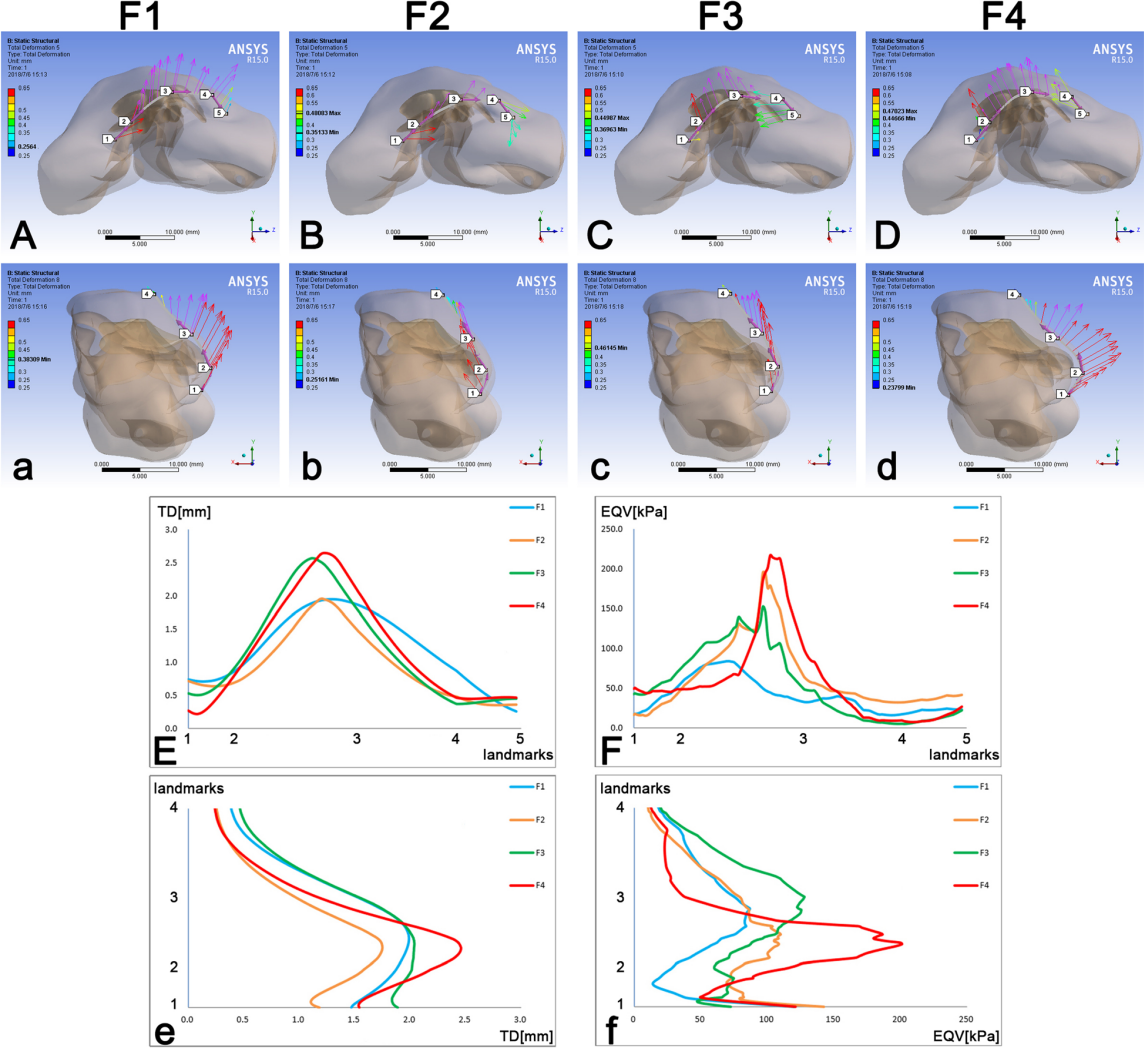
The TD and EQV at major landmarks on the skin envelope consequent to different force loadings. **A**, **B**, **C**, **D** The vectors of TD on Path one; **a**, **b**, **c**, **d** The vectors of TD on Path two; **E** The TD on Path one; **F** The EQV on Path one; **e** The TD on Path two; **f** The EQV on Path two. TD, total deformation; EQV, equivalent von-mises stress. The definitions of two paths were described in the legend of Fig. 2

Specific outcomes on two paths were also demonstrated. F1 moved Path one to the non-cleft side and moved Path two anteriorly (Fig. 5Aa). F2 moved Path one to the non-cleft side and posteriorly at the non-cleft-side alar base. Meanwhile, F2 could elevate the nose cranially but also enhance the collapse of the nasal tip a little (Fig. 5Bb). F3 moved Path one to the cleft side and moved Path two cranially (Fig. 5Cc). When loading F4, Path one was moved to the cleft side, and Path two was moved anteriorly, in which the collapse of the nasal tip was restored well (Fig. 5Dd).

The TD patterns of four forces on Path one were almost the same, all reaching the peak at the area near the nasal tip of the cleft side. F1 and F2 could cause a similar maximum deformation among Path one, but the TD varied more steadily when loading F1. The peak of F3 biased a bit to the cleft side compared to F4 (Fig. 5E). The patterns of EQV on Path one were as the same when loading F2, F3 and F4, in which the peak was at the area near the nasal tip of the cleft side. The EQV of F1, however, was demonstrated much lower than the other three forces on Path one (Fig. 5F). The TD and EQV patterns of four forces on Path two were almost the same, in which TD reached the peak around the nasal tip (Fig. 5e) as the maximum peaks of EQV varied from the nasal tip to the dorsum, while the minimum peaks varied from the columella base to the nasal tip (Fig. 5f).

## Discussion

Restoration of unilateral cleft lip nasal deformity was focused on repairing the pathological characteristics [12, 16, 18–20]. Various corrective procedures are designed, and the outcomes were all inspired. However, in the biomechanical dimension, it is formidable to decide which procedure is most appropriate for a cleft lip rhinoplasty.

Suspension suture, also named as elevating suture, has been widely applied to cleft lip rhinoplasty. The mechanisms of different suspension sutures, however, were poorly revealed. Such biomechanical analyses could be demonstrated, since the tissue displacement and stress distribution could be recapitulated by finite element method. Our latest work first established a finite element model of a unilateral cleft lip infant specimen basing on the micro-MRI imaging. This study suggested that three forces representing alar base adduction, columella straightening and nasal elevation should all be included in the cleft lip rhinoplasty [12]. Another work concentrated on the deformation and stress caused by two maneuvers during secondary cleft lip rhinoplasty, which attempted to reveal the correlation between the relapse and surgical techniques [11].

In this study, rhinoplasty processes were performed on our primary unilateral cleft lip model. The closure of cleft lip was first mimicked. Next, four shared suspension sutures were recapitulated: 1. Millard’s method: Medially, anteriorly and cranially directed force on the tip of medial crus (F1) to simulate the suture fastening both medial crura together [14]; 2. Cutting’s method: Medially, posteriorly and cranially directed force on the medial crus (F2) to simulate the suture which sewed both medial crura and the non-cleft-side upper lateral cartilage together [15]; 3. McComb’s method: Force directed to the nasal radix and paralleled to the dorsum on the intermediate crus (F3) to simulate the suture elevating the alar cartilage cranially [16]; 4. Noordhoff’s method: Anteriorly directed force on the intermediate crus (F4) to simulate the suture elevating the alar cartilage superiorly [17]. The total deformation represented the immediate surgical outcome. The distribution and magnitude of stress, in a certain degree, predicted the probability of relapse.

When closing the cleft, the deformation of the nasal structure was detected. The maximum deformation was demonstrated around two alar bases and predicted the function of alar base adduction. The deformation on Path one, except the alar base part of the cleft side, indicated the deviation of the columella was restored. The second largest deformation on Path one near the dome indicated that the collapse of the alar dome was also reconstructed (Fig. 2D). The explanation for the flat stress pattern near the dome could be responded to the structure and morphology of soft tissue near the dome (Fig. 2E). The closure of cleft lip was critical to alar base adduction as well as the restoration of columella deviation and alar dome collapse.

The deformation on the cartilage framework indicated that all forces could elevate the nasal tip (Fig. 3ABCD), and the stress on the framework always located at the force loading place (Fig. 3abcd). F3 triggered the most significant cranial movement of the alar cartilage on the cleft side which led to a wider stress distribution on the upper cartilage (Fig. 3Cc). F4 had little impact on the upper cartilage because it directed superiorly (Fig. 3Dd). F1 and F2 pushed the alar cartilage to the non-cleft side, which might enhance the asymmetry of the whole nasal structure (Fig. 3AB).

Forces on the cartilage resulted in the morphological change of the soft skin envelope. Four different forces, however, led to similar deformation region on the soft envelope (Fig. 4e, f, g, h), which indicated that the influence by moving alar cartilage at the cleft side was identical according to the anatomic location of the cartilage. Moreover, this outcome might also suggest that each part of the nasal framework performed their respective duties in rhinoplasty. The stress distribution on the surface of the skin envelope, on the other hand, possessed no obvious difference from different force (Fig. 4i, j, k, l).

Although these forces gave rise to similar quintessential deformation region, the outcomes were variable. F1 and F2 tended to move the whole nasal structure to the non-cleft side, which enhanced the nasal asymmetry. F1 could restore the collapse of nasal tip whereas F2 only elevated cranially (Fig. 4a, b). The reason for this phenomenon was that F2, the suture connecting the upper lateral cartilage, gave a posterior vector during this process. F3 and F4 had the function on restoring the symmetry as well as repairing the nasal tip projection (Fig. 4c, d).

The functions of each force on the horizontal and sagittal plane were well manifested by applying two paths on the skin envelope. According to the horizontal view of Path one, the asymmetry enhancement by F1 and F2 was demonstrated (Fig. 5AB), while the symmetric restoration was performed by F3 and F4 (Fig. 5CD). From the sagittal view, the nasal tip projection could be restored significantly by F1 and F4 (Fig. 5ad). F2 and F3 concentrated on elevating nasal tip cranially (Fig. 5bc).

F1, representing the suture which fastened two medial crura together, could be the most potential maneuver for cleft lip rhinoplasty. F4 indeed triggered the most significant deformation on the skin envelope, but it also led to a higher level than the other three forces (Fig. 5EeFf). F1, however, resulted in a stead and gentle deformation pattern on Path one which indicated a much more symmetrical change of the skin envelope (Fig. 5E). Meanwhile, F1 generated the lowest level of stress on Path one and two, which revealed a lower probability of relapse after surgery (Fig. 5Ff). To be more specific, the suspension maneuver proposed by Millard [14] could be the most positive to the primary unilateral cleft lip rhinoplasty according to the biomechanical outcomes. However, it was not fair to affirm the other three maneuvers were not suitable. For example, when facing collapsed nasal tip, the suspension maneuvers proposed by McComb and Noordhoff should be considered [16, 17]. Different direction of the nasal tip addition can be realized by these two methods respectively. Although we found that a high level of stress can be generated by these two methods, overcorrection should be the solution. For significant asymmetry of the nose with less nasal tip projection problem, the Cutting’s method could restore it easily without high stress remained in the nasal system, which could reduce the possibility of relapse [15].

Our study only tried to demonstrate the mechanisms of different suspension sutures basing on computational simulation. Meanwhile, these outcomes should be proven again in the future after the comparison of clinical outcomes from different surgical maneuvers. When performing a cleft lip rhinoplasty, a cleft surgeon should always bear the features of each maneuver in mind and try to apply the most beneficial practice.

This study shared the limitations among all theoretical models. The whole finite element analysis was only set in the elastic region [7, 11, 12], and the forces applied in this study were all based on trial and error, which was responded to the near-reality deformation that was generated on the skin envelope. The loading spot and direction of the force were also decided to base on the description of surgical maneuvers [14–17]. Meanwhile, it was difficult to define the accurate magnitudes of the force of sutures. The outcomes could be influenced due to the non-homogeneous property setting of human tissue. Multiple features which influenced the long-term stability of rhinoplasty were not fully recapitulated in this model due to technical limitations. First, the tension forces on the skin, which could be generated mostly by the scar contracture after surgery, was impossible to mimic due to the lack of information about the magnitude or direction of the contractile forces. Second, we simplified the model with no consideration for the subcutaneous dissection and periosteal scoring, which could make the model too complex for accurate calculation. The bony structure and the position of the nasal septum were not taken into consideration, which would influence the changing mechanism of the overlap nasal system. The closure of cleft lip was only simulated by loading force on the surface of the model which was simplified and was different from the real surgery. Moreover, although micro-MRI could help define the locations of the cartilages, it cannot reveal different muscles in this case, which could be the purpose for the future study.

## Conclusions

Each suspension suture had its characteristics respectively. The simulation suggested that the suture proposed by Millard which sewed both medial crura could be the most potential maneuver for cleft lip rhinoplasty, because it can symmetrically restore the shape of the nose without incurring a significant increase in stress. Meanwhile, the sutures of McComb and Noordhoff could be chosen according to the needed direction of nasal tip restoration with overcorrection for avoiding relapse. For asymmetry nose without any significant nasal tip problem, Cutting’s method could be applied. The future study will concentrate on the clinical outcomes comparison among those four common sutures based on the finite element simulations.

## Data Availability

All datasets or analyses during this study are included in this published article.
